# Behavioral Impairment in Aquatic Organisms Exposed to Neurotoxic Pollutants

**DOI:** 10.3390/toxics10050243

**Published:** 2022-05-10

**Authors:** Melissa Faria, Carlos Barata, Demetrio Raldúa

**Affiliations:** Institute for Environmental Assessment and Water Research (IDAEA), CSIC, Jordi Girona 18, 08034 Barcelona, Spain

Neuroactive chemicals are compounds that can modulate, at very low concentrations, the normal function of the central nervous systems of an organism through various primary modes of action (MoA). It has been estimated that around 13% of all detected chemicals in European Rivers have neuroactive potential [1]. This group of compounds includes pesticides (organophosphates, neonicotinoids insecticides, carbamates, organochlorines and pyrethroids), stimulants, CNS-acting pharmaceuticals (including, but not restricted, antidepressants, anxiolytics and antipsychotics) and illicit drugs. Globally, the use of neuroactive compounds is increasing due to growing of urban population. The development of modern chemical screening approaches have allowed for the detection and quantification of many of these chemicals in parallel, confirming the co-occurrence in mixtures of many chemicals with similar or different MoA, which in addition rises the concern about their potential combined effects. It is a known fact that such neuroactive chemicals affect wildlife behavior, with the prospective to cause detrimental effects on individual, population and community levels of ecological organization [2]. In this special issue on “Behavioral Impairment in Aquatic Organisms Exposed to Neurotoxic Pollutants”, original research and review articles addressing behavioral impairment induced by the exposure of different invertebrate and vertebrate aquatics species to neuroactive chemicals, are presented. In these studies, different methodological approaches are used, including multi-compartment systems, automated plug and play systems granting medium- and high-throughput screening, as well as, homemade setups systems. Furthermore, association to changes at lower levels of biological organization, such as, gene expression, biochemical activities, neurochemical signaling and macromolecules damage, are also described.

Aiming to increase our current understanding on the ecological and toxicological dimension of environmental occurrence of psychoactive pharmaceuticals in aquatic ecosystems, the review by Stumper and Margiotta-Casaluci 2022 [3] identified 210 CNS-acting pharmaceuticals currently prescribed in the UK. Through the analysis of the PHARMS-UBA database, authors found that presence of 84 of these pharmaceuticals had already been reported in surface waters around the world, of which 33 belong to the list of the 50 most prescribed in the region. Moreover, authors calculated the Predicted Environmental Concentrations (PECs) and then, using the Fish Plasma Model approach, the Predicted Fish steady state Plasma Concentration (F_ss_PC) for all the identified pharmaceuticals. By comparing F_ss_PC with the Human Therapeutic Plasma Concentration (HTPC), expressed a C_max_, authors estimated the Predicted Pharmacological Risk for each pharmaceutical. Finally, by using this approach, 32 of the pharmaceuticals were classified as exhibiting potential high and medium risk of eliciting pharmacological effects at their PECs in fish. The results presented in this review should be extremely useful to guide future research on the risk of the environmental risk of neuroactive chemicals in aquatic ecosystems.

When it comes to the quality of the information provided by different ecotoxicological approaches, all of them have their advantages and limitations. The review by Araújo et al. 2020 [4] discusses the limitations of traditional and standardized forced exposure approaches in predicting the ecological relevance of the presence of chemicals stressor in the environment. Authors emphasize that, whereas a force exposure approach considers that the environments are chemically homogeneous and that the option to avoid exposure is inexistent, the natural environment is clearly heterogeneous. Therefore, one must consider that when organisms are confronted with contaminants, three different reactions should be considered: conformity, regulation and avoidance. In this review, the need to integrate a more direct assessment of ecological implications of behavioral changes due to the presence of a chemical stressor is highlighted. The authors propose an approach in which Stress and Landscape Ecology could be integrated in order to better understand the true effect of a contaminant on the structure and function of an ecosystem. This would be possible by the combination of non-forced multi-compartment approach, also known as “avoidance method”, with the traditional and standardized forced exposure approach.

While there is widespread agreement that analysis of behavioral responses provides a more sensitive endpoint for assessing the environmental effects of neuroactive chemicals than lethality, environmental regulatory agencies have yet to include behavioral analysis among the endpoints to be analyzed in ecotoxicology. One assumption is that this may be due to the absence of optimized and standardized behavioral assays [5]. Aquatic invertebrates such as *Daphnia* spp. and *Artemia* spp. are commonly used model species to analyze different endpoints of ecotoxicological assessments, including behavior. Behavioral studies with such species have high-throughput potential, however methodological discrepancies make it difficult to be able to compare results from different studies. Two factors easily controlled that may have important implications on the response outcome are the arena size and light intensity. For *Artemia franciscana* a medium to large arena size (12 and 6 well plates) and not light intensity was crucial for a stable swimming speed response, indicating that there could be a compromise between increasing the throughput of the analyses and providing enough space for an even behavior [5].

Pharmaceuticals are a major emerging category of chemicals that pose real concern for the health of aquatic ecosystems. Invertebrate species play an essential role in the stability and well being of the ecosystems and are most threatened by the presence of these chemicals. The anti-depressant drug fluoxetine, at environmental relevant concentrations, increased swimming speed of *A. franciscana* [5]. On another study from this SI, the MoA of deprenyl was assessed for the first time in *Daphnia magna*. Deprenyl is a drug prescribed to treat major depressive disorders and Parkinson’s Disease, increasing serotonin signaling through inhibition of monoamine oxidase (MAO) [6], the enzyme responsible for its breakdown. Behavioral changes observed in *D. magna* exposed to deprenyl included low basal locomotor activity and reduction in the habituation light stimuli. Furthermore, *D. magna* exposed to deprenly exhibited inhibition of MAO-activity and a concomitant increase in the serotonin and dopamine levels, suggesting the presence of vertebrate MAO-like activity in this species. Finally, as proof of concept, behavior and molecular changes caused by deprenyl were found contrary with those observed for serotonin antagonistic drug, 4-Chloro-DL-phenylalanine (PCPA).

An analogous study was executed using *Danio rerio* (zebrafish) larvae exposed to the serotonin signaling stimulants deprenyl and fluoxetine and to the serotonin synthesis inhibitor PCPA [7]. Similar behavioral outcomes were observed for both anti-depressants, including hypolocomotion, reduced escape responses evoked by vibrational and visual stimuli and increased habituation to the vibrational stimuli, which contrasted with those observed for PCPA. At lower levels of organization, deprenyl’s effect was more potent, abolishing MAO activity, downregulating serotonin synthesis and transporter genes and augmenting serotonin and dopamine levels. Moreover, co-exposure of opposed serotonin signaling drugs revealed full recovery of several impaired responses. It is also interesting to highlight the homology of responses observed between *D. magna* and *D. rerio* to acute deprenyl exposure.

It is a well-known that the developing brain is more sensitive to the effect of chemicals than the adult brain [8], and that developmental exposure may result in subtle effects but can have a profound impact when amortized across the life span of and organism, permanently altering normal biological processes, which can be reflected in the organism behavior. When testing new chemicals, conventional OECD toxicity tests may not reflect their true hazardous impact to organism in the natural environment, therefore multiple experimental approaches should be applied for proper risk assessment. Anti-fugal natural extracts *Equisetum arvense*, *Mimosa tenuiflora* and Thymol, are suggested as a safer alternative for synthetic fungicides. Zebrafish developmental exposure to sublethal concentrations, up to 200 times lower than the reported 50% lethal concentrations (LC50s), showed that the first two extracts could be safe to use due to mild or absence of biological significance, however, Thymol showed to be lethal, teratogenic, alter antioxidant defenses and induce fear- and anxiety-like disorders in zebrafish eleutheroembryos [9].

Risk assessment of chemicals is usually conducted for individual chemicals whereas mixtures of chemicals occur in the environment. The different combinations of chemicals are associated with significantly different effects on communities of aquatic ecosystems. Considering that neuroactive chemicals are a group of contaminants that dominate the environment, it is then imperative to understand the combined effects of mixtures [10]. The commonly used models to predict mixture effects, namely concentration addition (CA) and independent action (IA), are thought to be suitable for mixtures of similarly or dissimilarly acting components, respectively. Furthermore, CA and IA models may be used to evaluate observations as antagonistic (less effect than predicted) or synergistic (higher effect than predicted). However, these predictions are mainly based for survival as endpoint, and it is unclear whether they can be implemented for mixture studies addressing behavioral endpoints. One challenge for the application of these predictive models (CA and IA), is that not always neuroactive substances based on similar MoA may have similar behavioral responses, so the question leis whether these models can be used to predict combined effects for neuroactive chemicals mixtures with different MoA but similar behavioral responses. Another issue that rises is whether chemicals with opposing effects can be predicted as antagonistic. In this special issue, Ogungbemi et al. 2021 [10] addressed these questions by investigating the effect over zebrafish embryos spontaneous tail coiling (STC) following exposure to mixtures of pesticides with different MoAs. Indeed, authors found that neuroactive substance with different MoA, such as propafenone and abamectin as well as chlorpyrifos and hexaconazole giving a similar direction of response outcome (hyper- or hypoactivity) seemed to be additive and therefore could be predicted using the CA and IA models. On the other hand, results that showed mixtures with both hyper- and hypoactivity-inducing components lead to an antagonistic interaction, and therefore, to qualitatively predict mixture outcomes of multi-complex mixtures as well as to understand deviations form additivity, the authors recommend considering information on common adverse outcomes of the chemicals.

Another approach to assess effects of mixtures of neuroactive chemicals, is to use their recorded concentrations in the environment. Santos et al. 2021 [11] analyzed the impact of mixtures of relevant concentrations of three common pesticides, glyphosate, chlorpyrifos and copper, over developmental stages of rainbow trout (*Oncorhyncus mykiss*). The authors found antagonistic effects over fish swimming activity when exposed to chemicals with opposing effects. It was suggested that the presence of copper and chlorpyrifos could have antagonized or reduced the effects of glyphosate on larvae swimming activity. When looking at responses at lower levels of organization, authors found additive or synergistic effects of the joint action of these pollutants, interestingly, the observed upregulation of genes involved in detoxification, mitochondrial metabolism and DNA repair suggested an adaptive response triggered to deal with toxic exposure.

In summary, this collection of original research and review works provides vital and updated information regarding research and challenges on behavior ecotoxicity of invertebrate and vertebrate aquatic organisms as well as the molecular mechanisms behind the effects.
